# Design and Synthesis of an Artificial Perpendicular Hard Ferrimagnet with High Thermal and Magnetic Field Stabilities

**DOI:** 10.1038/s41598-017-16761-z

**Published:** 2017-12-05

**Authors:** Jun Lu, Siwei Mao, Xupeng Zhao, Xiaolei Wang, Jian Liu, Jianbai Xia, Peng Xiong, Jianhua Zhao

**Affiliations:** 1 State Key Laboratory of Superlattices and Microstructures, Institute of Semiconductors, Chinese Academy of Sciences, P.O. Box 912, Beijing, 100083 China; 2 College of Materials Science and Opto-Electronic Technology, University of Chinese Academy of Sciences, Beijing, 100049 China; 3 Department of Physics, Florida State University, Tallahassee, FL 32306 USA

## Abstract

It is of great fundamental and practical interest to develop effective means of modulating the magnetic hystereses of magnetic materials and their heterostructures. A notable example is the exchange bias (EB) effect between an antiferromagnet or ferrimagnet and a ferromagnet, which has been widely employed to manipulate magnetic anisotropy in spintronic devices and artificial magnets. Here, we report the design, synthesis and characterization of a synthetic perpendicularly-magnetized ferrimagnet based on [Mn_2.9_Ga/Co_2_MnSi]_n_ superlattices, which attains thermal stability above 400 K and a coercive field up to 45 kOe through a mechanism of magnetic compensation. The structure is incorporated into a prototype Heusler alloy and MgO barrier based magnetic tunnel junction, which demonstrates high dynamic range linear field responses and an unusual in-plane EB effect. With increasing temperature, the coercive field reaches beyond 70 kOe at 400 K in this device due to the increasing degree of magnetic moment compensation in the superlattice. The results demonstrate that the compensation mechanism can be utilized to achieve simultaneous thermal robustness and high coercivity in realistic spintronic devices.

## Introduction

The magnetic hysteresis is a signature characteristic of a ferromagnet and the basis for most of its applications. The abilities to control the magnitude and position of the coercive field of a ferromagnet are critical for a variety of magnetic material and device functionalities. A prominent example is the exchange bias (EB) effect^1^, which is ubiquitous at antiferromagnet/ferromagnet (AFM/FM) and hard ferrimagnet/ferromagnet (FIM/FM) interfaces. EB is widely employed to produce unidirectional magnetic anisotropy in AFM/FM or FIM/FM bilayers; it shifts the magnetic hysteresis loop of the FM, resulting in an increase or decrease of the coercive field at either side of the hysteresis. EB has been utilized to engineer artificial hard magnets free of rare earth elements^2^, and to produce a pinned FM layer in spin valves and magnetic tunnel junctions (MTJs)^3–5^. For the latter, the EB in an AFM/FM bilayer is often used in combination with the exchange coupling in an artificial AFM, e.g. in Co/Ru(Cu)/Co/IrMn^6^, in order to minimize the magnetostatic interactions in spin-valve and MTJ devices, and to amplify the exchange bias field significantly beyond the typical values in AFM/FM bilayers^4^. Besides the AFM/FM heterostructures, EB is also widely observed in FIM/FM hybrid structures^7–11^. The FIM/FM systems offer an additional advantage that both the coercivity and EB field may be significantly modulated via the compensation state of the FIM^12,13^. However, the EB field is normally below a few kOe in AFM/FM, while the thermal stability is often poor in FIM/FM structures. More recently, ferromagnets with large perpendicular magnetic anisotropy (PMA) have gained increasing relevance in high-density magnetic recording media and spin transfer torque magnetic random access memory (STT-MRAM), due to their superior thermal and magnetic stability over their in-plane magnetized counterparts. Therefore, perpendicularly-magnetized compensated FIMs with near zero net magnetization, such as Sm_0.974_Gd_0.026_Al_2_^14^, DyCo_4_^12^, DyCo_5_^15^, TbCo^13^, TbFe^16^, DO_3_-Mn_3_Ga^17^ and Mn_2.4_Pt_0.6_Ga^18^, have attracted much recent attention, and they have been used as the base layer for creating perpendicularly-magnetized FIM/FM bilayers including SmAl_2_/Sm_0.974_Gd_0.026_Al_2_^7^, DyCo_5_/Ta/Fe_76_Gd_24_^9^, [Co/Ni]_n_/TbCo^10^, TbFe/[Co/Pt]_n_^11^. However, there are a couple of important drawbacks with these materials. The first is the low compensation temperatures, which would limit the utility in practical applications at room temperature or higher. And, with the exception of the compensated Heusler alloys (e.g., the ideal DO_3_-Mn_3_Ga), the spin polarization is very low, which essentially precludes *direct* use of these perpendicularly-magnetized compensated FIMs as a spin polarized electrode^19^. Therefore, a pertinent question is: Is it possible to design and synthesize an artificial FIM which simultaneously exhibits PMA, high spin polarization, very large coercivity, and superior thermal stability?

Inspired by the compensated FIM compounds and artificial AFMs, we propose a perpendicular hard artificial FIM based on AFM-coupled [FM1/FM2]_n_ superlattices, whose compensation temperature can be flexibly tuned by the thickness ratio of FM1/FM2. Previously, a superlattice FIM of [MnGa/Co_2_FeAl]_n_ was reported by Q. Ma *et al*., and the magnetic properties were tuned by adjusting the thickness of MnGa layer^20^. Here, we realized a thermally robust, perpendicular hard artificial FIM based on perpendicularly-magnetized, AFM-coupled [Mn_2.9_Ga/Co_2_MnSi]_n_ superlattices (we describe the form of Mn_2.9_Ga as MnGa for simplicity from the outset). The mechanism of the AFM coupling in this type of MnGa/Co_2_MnSi (Co_2_FeAl) bilayers may originate from an AFM-coupled MnGa/Co interface rooted in the Pauli exclusion effect^20–22^. MnGa is a perpendicularly magnetized ferromagnet with a large magnetocrystalline anisotropy (*K*_u_) up to 21.7 Merg/cm^3^, and tunable saturation magnetization (*M*_s_) via variation of the composition and/or growth conditions^23,24^. An ultralow damping constant of 0.008 was determined from optical pump-probe measurement^25^, and a spin polarization of 58% as well as a Curie temperature of 730 K was also observed^26,27^. These features make it an attractive candidate for modern magnetic information storage devices at sub-10 nm nodes. On the other hand, Co_2_MnSi is a well-known Heusler alloy with 100% spin polarization and a high Curie temperature near 1000 K^28^; it has been widely studied for applications in spin valve and MTJ structures^29–31^. Recently, MnGa/Co_2_MnSi bilayers were synthesized and the exchange coupling was studied^32,33^. Although at a thickness of 20 nm the Co_2_MnSi exhibits *in-plane* magnetic anisotropy, in an applied perpendicular magnetic field higher than its demagnetization field, perpendicular AFM coupling between MnGa and Co_2_MnSi was observed. Here, by reducing the Co_2_MnSi thickness to 1 nm, we successfully realized AFM-coupled [MnGa/Co_2_MnSi]_n_ superlattices with full PMA, which presents a model system of perpendicularly-magnetized hard FIM with near zero net magnetization at tunable temperatures. We demonstrate that by properly setting the compensation point, a very high perpendicular coercivity is realized at room temperature, and it continues to *increase* up to 45 kOe *with increasing temperature* up to 400 K, which is opposite to the temperature dependence in a conventional FM. The compensation temperature is limited by the individual Curie temperatures of the AFM-coupled FM layers, hence the [MnGa/Co_2_MnSi]_n_ superlattices have the potential for achieving a compensation temperature much higher than 400 K. Furthermore, we incorporate the [MnGa/Co_2_MnSi]_n_ into a prototypical MTJ stack of GaAs/Co_2_MnSi/ [MnGa/Co_2_MnSi]_n_/MgO/CoFe/Pd, which yields an appreciable tunneling magnetoresistance (TMR) of 31% at room temperature. With increasing temperature, the coercive field reaches beyond 70 kOe at 400 K in this device due to the increasing degree of magnetic moment compensation in the superlattice. The results demonstrate that the compensation mechanism can be utilized to achieve simultaneous thermal robustness and high coercivity in realistic spintronic devices.

## Results

### Design of perpendicular hard AFM-coupled [FM2/FM1]_n_ FIM superlattices

**Figure 1:**
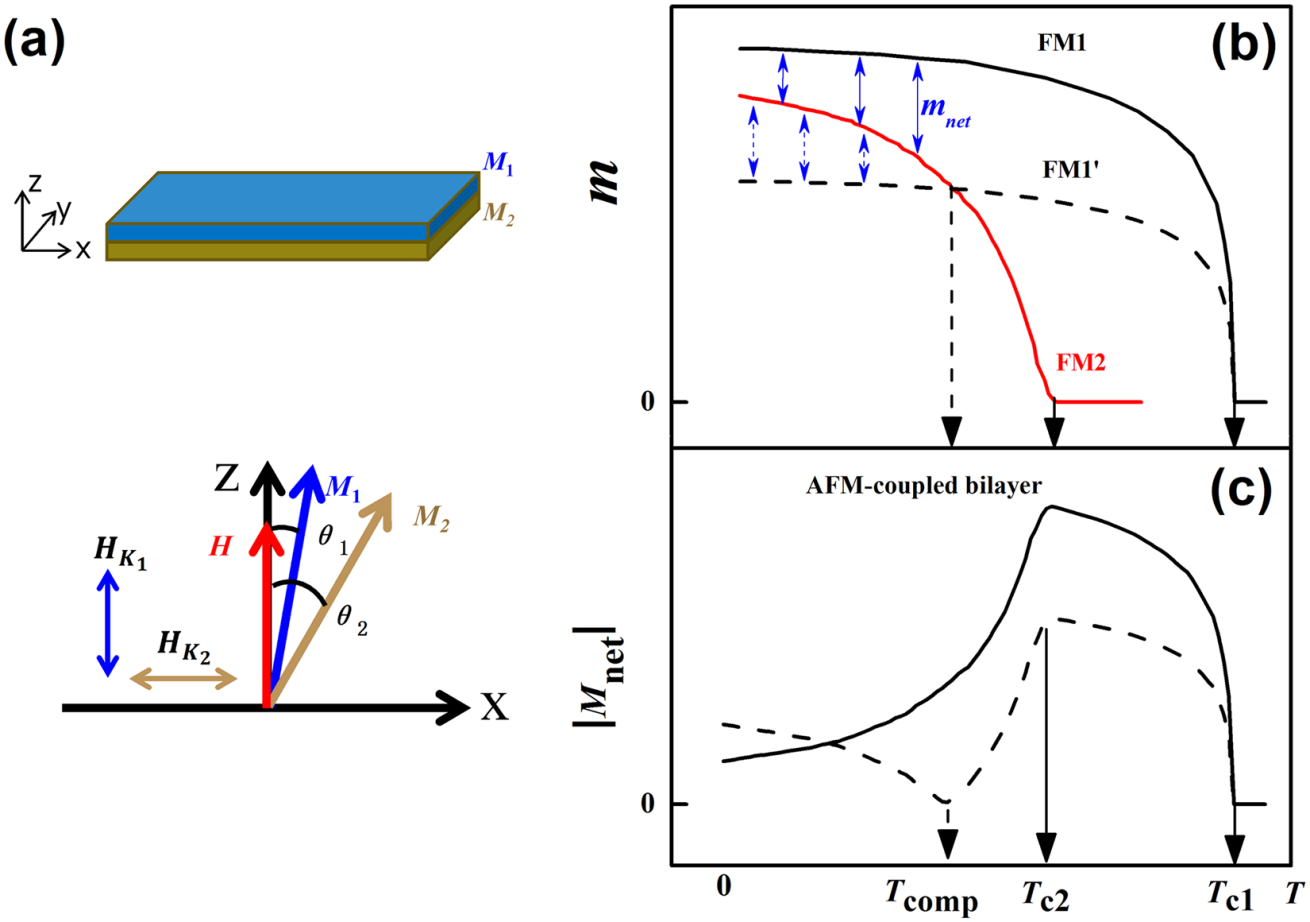
Analytical model and principal compensation mechanism in AFM-coupled bilayers. (**a**) Schematic drawing of an FM1/FM2 AFM-coupled bilayer structure, and magnetization *M*_1_ (*M*_2_) and magnetic anisotropy field *H*_K1_ (*H*_K2_) directions of FM1 (FM2). (**b**) Schematic diagram depicting different scenarios of magnetic moment (*m*) versus temperature (*T*). The AFM-coupled ferromagnetic films FM1 and FM2 have Curie temperature *T*_c1_ and *T*_c2_ respectively. When *m* of FM1 is greater than that of FM2 (solid line), there is no intersection point between the two *m*-*T* curves. At a smaller *m* of FM1, the *m*-*T* curve of FM1 (dash line) will cross the *m*-*T* curve of FM2 at a compensation temperature of *T*_comp_. (**c**) The two scenarios in (**b**) result in two distinct *M*_net_ - *T* curves at temperature below *T*_comp_: One has decreasing *m* with increasing *T*, and the other is opposite.

Figure 1 (a) depicts an analytical model for the AFM-coupled FM1/FM2 bilayer system. In this structure, the thickness of each individual layer is smaller than the exchange length, and the soft ferromagnetic film FM2 is strongly AFM-coupled with the PMA hard ferromagnetic film FM1. The total free energy per unit area of a bilayer system can be expressed as

1 $$\begin{array}{c}E={K}_{1}{t}_{1}{\sin }^{2}{\theta }_{1}+{K}_{2}{t}_{2}{\cos }^{2}{\theta }_{2}-{M}_{1}{t}_{1}H\,\cos \,{\theta }_{{\rm{1}}}-{M}_{2}{t}_{2}H\,\cos \,{\theta }_{{\rm{2}}}\\ \,\,\,\,+2\pi {M}_{1}^{2}{t}_{1}{\cos }^{2}{\theta }_{1}+2\pi {M}_{2}^{2}{t}_{2}{\cos }^{2}{\theta }_{2}-{J}_{ex}\,\cos ({\theta }_{1}-{\theta }_{2}),\end{array}$$

where *K*_1_ is the perpendicular anisotropy of the perpendicular hard magnet FM1, *K*_2_ is the in-plane uniaxial anisotropy of the soft magnet FM2, *M*_1_ (*M*_2_) and *t*_1_ (*t*_2_) are the saturation magnetization and thickness of FM1 (FM2) respectively, *θ*_1_ (*θ*_2_) is the angle between *M*_1_ (*M*_2_) and the film normal, and *J*_ex_ is the exchange coupling constant between *M*_1_ and *M*_2_. Magnetic field *H* is applied perpendicular to the film plane. For very strong AFM coupling, the exchange coupling field is much larger than the out-of-plane demagnetizing field of FM2 and the coercive field of FM1, so there is a simple relation *θ*_2_ = *θ*_1_ − π. By setting $\partial E/\partial {\theta }_{1}=0$, and *θ*_1_ = 0, we obtain the coercive field of the bilayer

2 $${H}_{c}\,=-2{K}_{{\rm{eff}}}/{M}_{{\rm{net}}},$$

where

3 $${M}_{{\rm{net}}}=({M}_{1}{t}_{1}-{M}_{2}{t}_{2})/({t}_{1}+{t}_{2}),$$

and

4 $${K}_{{\rm{eff}}}=({K}_{1}{t}_{1}-{K}_{2}{t}_{2})/({t}_{1}+{t}_{2})-2\pi ({M}_{1}^{2}{t}_{1}+{M}_{2}^{2}{t}_{1})/({t}_{1}+{t}_{2}).$$

This indicates that the coercive field of the AFM-coupled bilayer could be effectively tuned by changing the net magnetization (*M*_net_) via the thickness ratio of FM1/FM2. Figure 1b,c) depicts schematically the temperature-dependent magnetic moment (magnetization) of the individual FM layers (the AFM-coupled bilayer). FM1 and FM2 have different Curie temperatures of *T*_c1_ and *T*_c2_. As illustrated in Fig. 1b and c, depending on the thickness ratio, there are two distinct net magnetization (*M*_*net*_) states with different temperature dependencies. In the first state, the *m*-*T* curves for FM1 and FM2 have no intersection point, so the *M*_net_ *increases* with increasing temperature. Upon decreasing the thickness of FM1, the two *m*-*T* curves cross at an intersection point *T*_comp_, and the *M*_net_ *decreases* with increasing temperature up to *T*_comp_. For a strongly AFM-coupled FM1/FM2 bilayer, its coercivity is mainly determined by *K*_eff_/*M*_net_. Therefore, for the case of a bilayer or superlattice in which *M*_net_ decreases with increasing temperature, the coercivity would *increase* with increasing temperature, which is opposite of the behavior of a conventional FM. By choosing a suitable FM1/FM2 thickness ratio and setting a proper compensation temperature *T*_comp_, one could obtain an artificial perpendicularly-magnetized FIM, in which the coercive field increases with increasing temperature.

### Magnetic properties of superlattices of [MnGa/Co_2_MnSi]_n_

**Figure 2:**
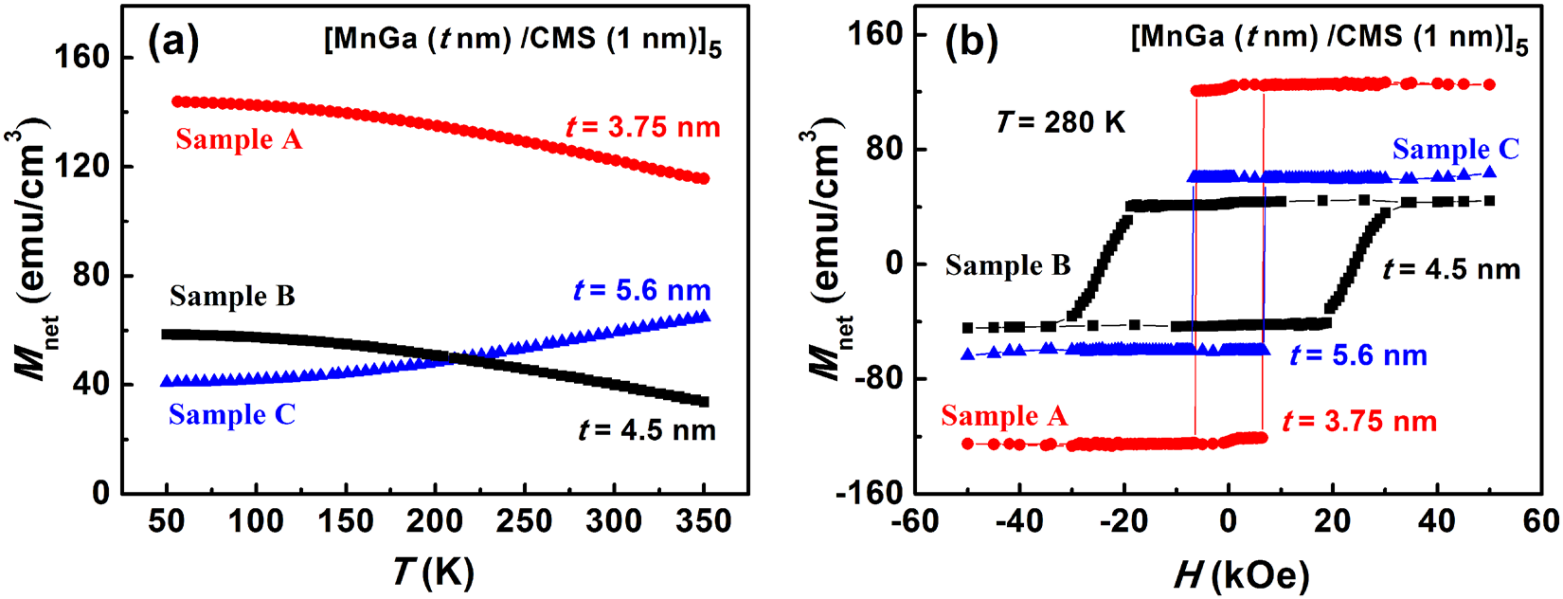
Magnetic properties of [MnGa (*t* nm)/Co_2_MnSi]_5_ superlattices. (Sample A: *t* = 3.75 nm, red circles; sample B: *t* = 4.5 nm, black squares; sample C: *t* = 5.6 nm, blue up triangles). (**a**) Net magnetization (*M*_net_) versus temperature (*T*) for samples A, B and C from 50 to 350 K. (**b**) Hystereses of samples A, B and C measured in a magnetic field range from −50 to +50 kOe at 280 K.

We have grown three samples of [MnGa/Co_2_MnSi]_n_ superlattices by molecular-beam epitaxy (MBE). For all three samples, the thickness of the Co_2_MnSi layer is fixed at 1.0 nm, while the MnGa thicknesses are chosen as 3.75, 4.5, and 5.6 nm. The number of period is *n* = 5. The temperature dependence of *M*_net_, and the magnetic hysteresis at 280 K were measured by a superconducting quantum interference device (SQUID) magnetometer. As shown in Fig. 2(a), for the sample with the thickest MnGa layer of 5.6 nm (sample C), there is no compensated state in the entire measurement temperature range, and the *M*_net_ increases with increasing temperature, consistent with the first case depicted in Fig. 1(a). As the MnGa thickness decreases to 4.5 nm (sample B), the *M*_net_ (*T*) shows qualitatively different behavior: The *M*_net_ decreases with increasing temperature, indicating the existence of a compensated state at a higher temperature. Further decreasing the thickness of MnGa to 3.75 nm (sample A), the ${M}_{{\rm{net}}}$ shows similar temperature dependence as that of sample B, but has a higher value at the same temperature. It should be noted that the results in Fig. 2a are consistent with a Curie temperature for the ultrathin (1 nm) Co_2_MnSi layer much reduced from its bulk value, to lower than that of MnGa, because if the Curie temperature of MnGa is lower than that of Co_2_MnSi, the crossover of the *m*(*T*) curves should occur larger, rather than smaller, MnGa thicknesses, which is contrary to the experimental data. Consequently, the compensation state was achieved in the optimal temperature range in sample B. Figure 2b shows the hystereses for the three samples at *T* = 280 K (the hysteresis loop of a single MnGa layer is shown in the section I of Supplementary Information for comparison). Two important aspects of the data are worth noting. First, although the magnetization dynamics of a hard ferromagnet is expected to be controlled primarily by the internal nucleation or pinning processes, the coercivity of sample B, which has the smallest ${M}_{{\rm{net}}}$ at room temperature, shows a marked enhancement over those of the other two samples. Second, despite that sample C has a much smaller *M*_net_ than sample A at the same temperature, they have essentially the same coercivity. These observations imply that the magnetization dynamics of the AFM-coupled superlattices can be controlled by the degree of compensation thanks to the lowered system Zeeman energy, as long as the thickness of each layer is smaller than a critical value. In these samples, although the Co_2_MnSi thickness is always smaller than its critical length; if the MnGa thickness is larger than the critical value, the magnetization dynamics will be similar to that of a single layer MnGa film even if the net magnetic moment of the bilayer is close to the compensation point. The critical length should be related to the exchange length, which is defined by $\sqrt{\frac{A}{{K}_{{\rm{eff}}}}}$, where *A* is the exchange stiffness and *K*_eff_ is the effective anisotropy constant. For MnGa, we assume *A* and *K*_eff_ have typical values of 10 pJ/m and 1 MJ/m^3^, respectively, resulting in an exchange length of 3 nm. For the superlattices of [MnGa/Co_2_MnSi]_5_, the thickness of the MnGa layer should be much less than 6 nm considering the double interfaces for each MnGa layer. Therefore, the temperature dependence of the coercive field for the three samples provides a model system to ascertain the effects of the magnetic compensation mechanism in the AFM-coupled superlattices.

**Figure 3:**
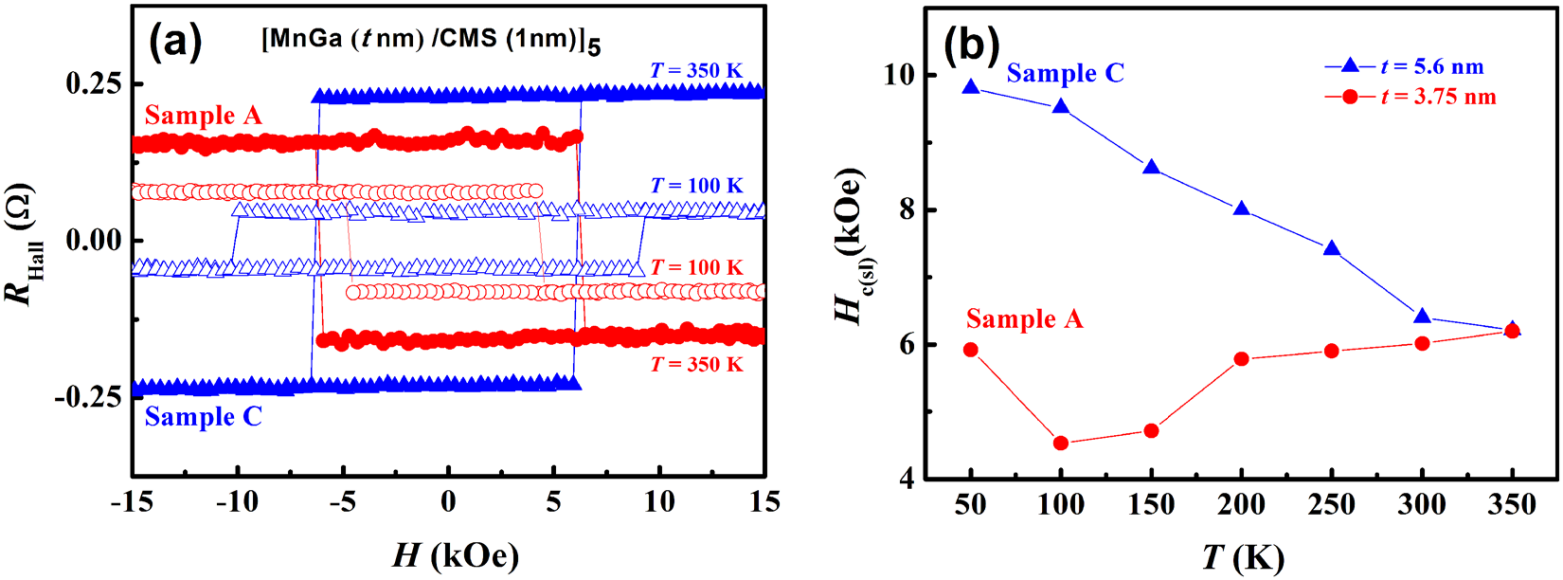
Hall resistance at different temperatures for [MnGa (*t* nm)/Co_2_MnSi]_5_ superlattices. (Sample A: *t* = 3.75 nm, red circles; sample C: *t* = 5.6 nm, blue up triangles). (**a**) *R*_Hall_ for samples A and C at two representative temperatures of 100 K (open) and 350 K (solid) in a magnetic field range from −85 to + 85 kOe. (**b**) Coercivity of superlattices versus temperature (*H*_c(sl)_ − *T*) curves from 50 to 350 K.

As one approaches the compensation temperature, it becomes increasingly challenging to measure the magnetic hysteresis and determine the coercivity of a compensated FIM by SQUID magnetometry. Fortunately, for our systems, the anomalous Hall effect (AHE) coefficients of MnGa and Co_2_MnSi are different and of opposite signs^34,35^. Consequently, for the MnGa dominated sample C, the AHE coefficient is positive, while for the Co_2_MnSi dominated sample A, it is negative, as shown in Fig. 3a. It is well established that the AHE signal is directly proportional to the magnetization of the sample^36^. Therefore, the temperature dependence of the coercivity of these superlattices can be measured by the AHE precisely as the temperature increases all the way to the compensation point. Figure 3a shows the Hall resistance (*R*_Hall_, in which the AHE makes the predominant contribution here) loops for samples A and C at two representative temperatures, and Fig. 3b shows the resulting coercivity *H*_c(sl)_ for the two samples at various temperatures. For the MnGa dominated sample C, the temperature dependence of the *H*_c(sl)_ is similar to that of a single MnGa layer; in contrast, for the Co_2_MnSi dominated sample A, its temperature dependence is not monotonic, and for temperatures higher than 100 K, the *H*_c(sl)_ actually *increases* with increasing temperature. At 50 K, the nucleation/pinning mechanism is likely to be dominant and also leads to enhancement of *H*_c(sl)_. These features are consistent with the SQUID measurements and the conclusion that the magnetic compensation modulates the magnetization dynamics in the thinner samples.

**Figure 4:**
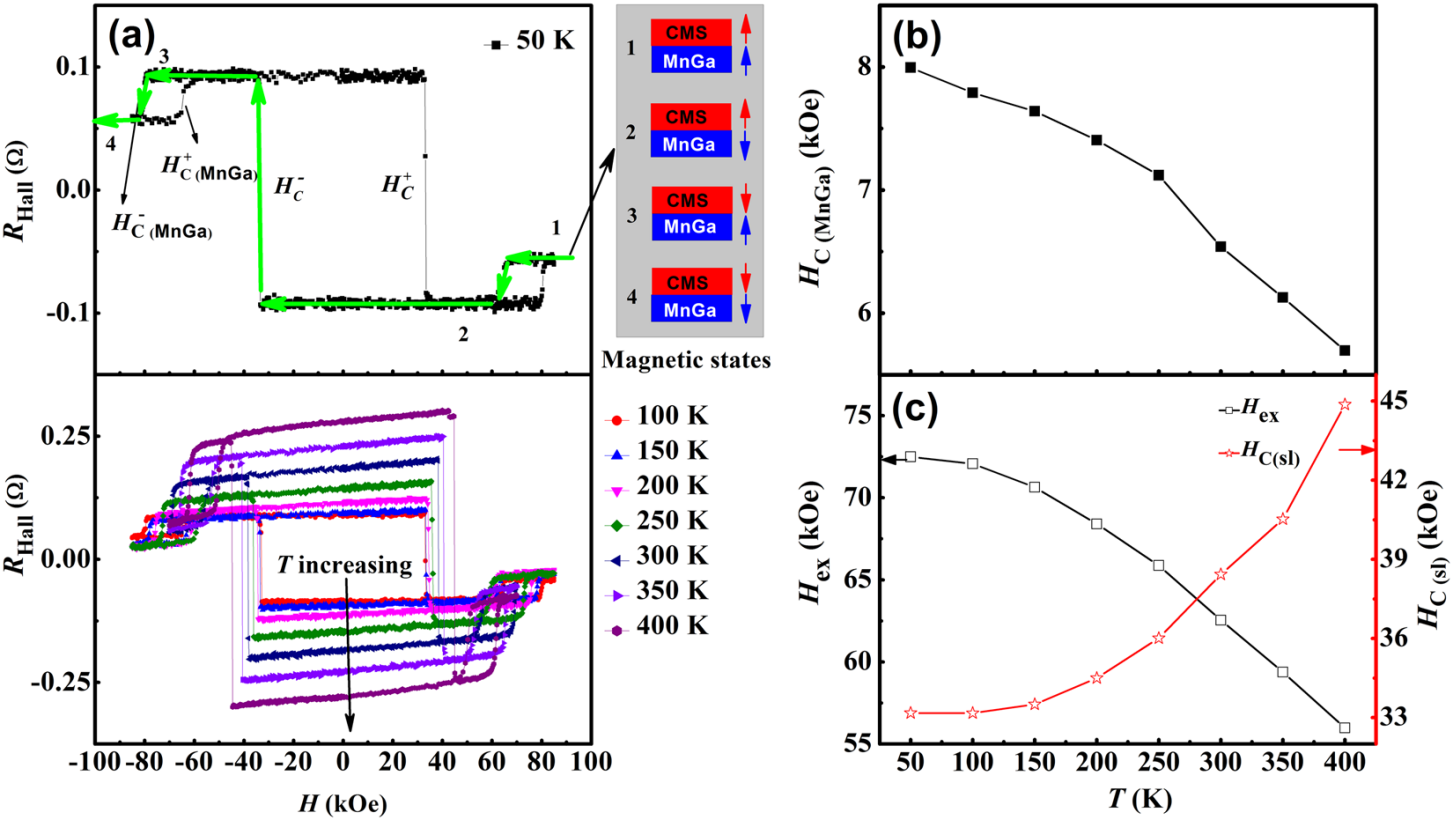
Hall resistance at different temperatures for [MnGa(*t* nm)/Co_2_MnSi]_5_ superlattice (sample B, *t* = 4.5 nm). (**a**) The *R*_Hall_ curves at different temperatures from 50 to 400 K. Four different magnetic states are depicted (red arrows for Co_2_MnSi, blue arrows for MnGa), and the transitions between them are indicated by numbers from 1 to 4 for a down-sweep (positive to negative: the green line with arrows). ${H}_{{\rm{C}}}^{-}$ and ${H}_{{\rm{C}}}^{+}$ are the coercive fields of the superlattice as a whole. ${H}_{{\rm{C}}({\rm{MnGa}})}^{+}$ and ${H}_{{\rm{C}}({\rm{MnGa}})}^{-}$ are the coercive fields for the MnGa layers biased by the exchange coupling field from the Co_2_MnSi layers in the superlattice. (**b**) The coercivity of the MnGa layers, determined by ${H}_{{\rm{c}}({\rm{MnGa}})}=\,({H}_{{\rm{C}}({\rm{MnGa}})}^{+}-{H}_{{\rm{C}}({\rm{MnGa}})}^{-})/2$. (**c**) The exchange coupling field ${H}_{{\rm{ex}}}[{H}_{{\rm{ex}}}=({H}_{{\rm{C}}({\rm{MnGa}})}^{+}+{H}_{{\rm{C}}({\rm{MnGa}})}^{-})/2]$ and the coercivity of the superlattices ${H}_{{\rm{c}}({\rm{s}}{\rm{l}})}[{H}_{{\rm{c}}({\rm{s}}{\rm{l}})}=({H}_{{\rm{C}}}^{+}-{H}_{{\rm{C}}}^{-})/2]$ as functions of temperature.

For sample B, with MnGa thickness of 4.5 nm and closest to the compensation point at high temperatures, the anomalous Hall resistances show an interesting new feature. As is evident in Fig. 4a, there are two hystereses attached to each Hall resistance sweep, suggesting two distinct magnetic switchings in the superlattice. The opposite signs of the AHE coefficients for MnGa and Co_2_MnSi provide a rare situation in which the coercivity of the superlattice (*H*_c(sl)_), the exchange coupling field between MnGa and Co_2_MnSi (*H*_ex_), and the coercivity of the MnGa (*H*_c(MnGa)_) can be uniquely determined from the switching fields in the Hall traces. The schematic diagram accompanying Fig. 4a shows four different magnetic states (red arrows for Co_2_MnSi, and blue arrows for MnGa); the transitions between these states are marked by numbers from 1 to 4 for the down-sweep (positive to negative). In the low field range, the AFM-coupled MnGa and Co_2_MnSi produce anomalous Hall voltages of the *same* sign, and switch together as the applied magnetic field increases to *H*_c(sl)_ (from state 2 to 3). As the applied magnetic field further increases, the magnetic moment of the MnGa layers switches from being antiparallel to parallel with the magnetic field (from state 3 to 4), and their contributions to the anomalous Hall voltage switch sign, leading to a decrease in the AHE signal. The AHE was measured at different temperatures from 50 to 400 K, which yields *H*_c(MnGa)_, *H*_ex_, and *H*_c(sl)_ at these temperatures, as plotted in Fig. 4b and c. *H*_c(MnGa)_ and *H*_ex_ show the normal trend, decreasing from 8.0 to 5.7 kOe and 72.5 to 56.0 kOe respectively from 50 to 400 K. However, in the same temperature range, the coercivity of the superlattice as a whole (*H*_c(sl)_) *increases* significantly from 33 to 45 kOe, and the rate of increase appears to accelerate as the compensation point is approached. In contrast, the enhancement is much weaker for sample A with thinner MnGa layers, implying that the compensation mechanism in the compensated natural FIM compounds is effective in this synthetic AFM-coupled superlattice. The perpendicular magnetic anisotropy *K*_1_ was deduced to be about 1 Merg/cm^3^ by a linear fitting of the *H*_c_ ∝ *K*_eff_/*M*_net_ curve. This implies that the hysteresis loop of this superlattice can be described by the Kronmüller equation, where the nucleation and pinning processes are two determining factors for the coercive field. The outstanding characteristics of this artificial FIM promise a variety of applications in artificial hard magnets, high density magnetic recording and, in particular, because of the high spin polarization of Co_2_MnSi, it may be used directly as a reference layer in perpendicular MTJs without the use of AFM EB pinning.

### Synthesis and characterization of MTJ incorporating [MnGa/Co_2_MnSi]_n_ synthetic FIM

**Figure 5:**
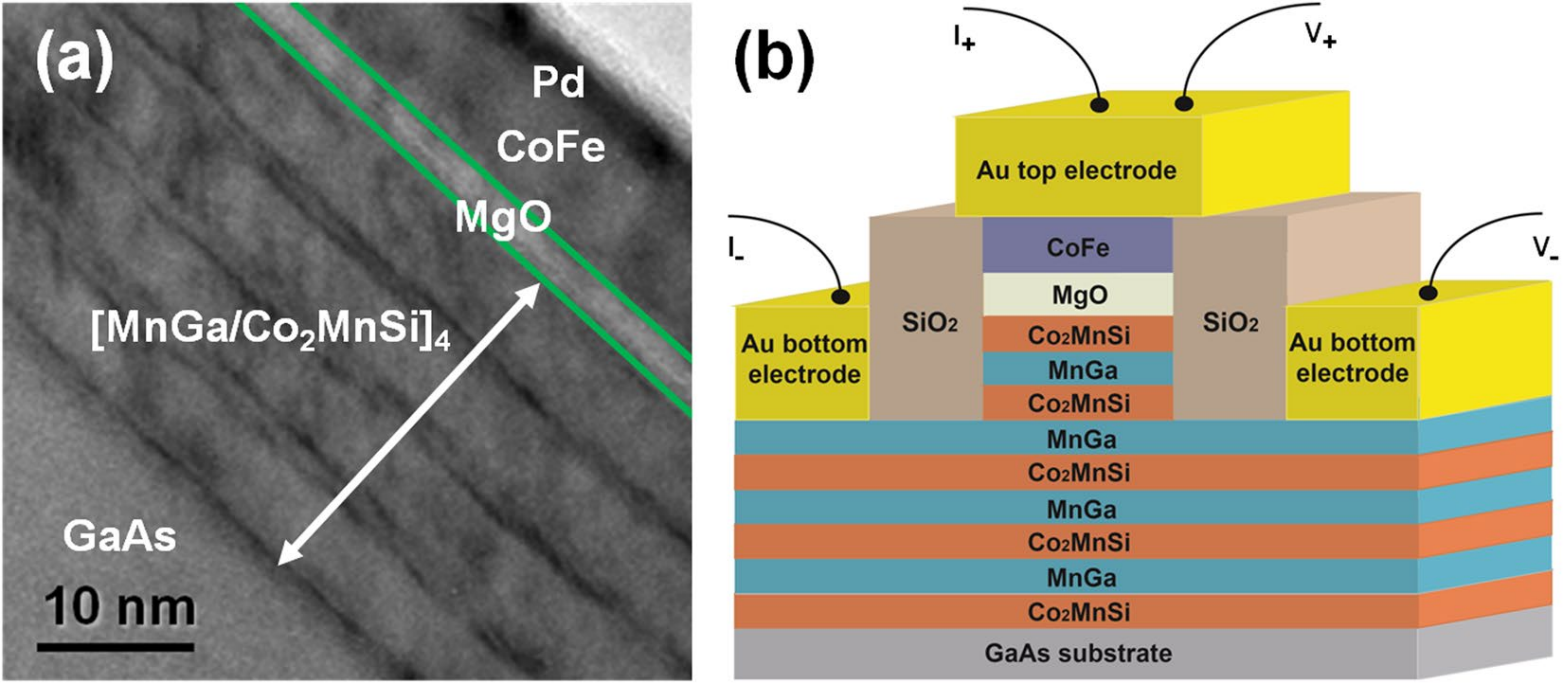
HRTEM image of the MTJ stack and a schematic of the MTJ device structure. (**a**) Transmission electron microscopy image showing the sharp interfaces between GaAs, [MnGa/Co_2_MnSi]_4_ superlattice, MgO and CoFe layers. Each layer is readily distinguishable. (**b**) Schematic of the prototype MTJ device with a [MnGa/Co_2_MnSi]_4_ superlattice as a reference layer, and CoFe as a sensing layer.

To demonstrate the efficacy of the MnGa/Co_2_MnSi synthetic FIM as the reference electrode of an MTJ, we have fabricated an epitaxial heterostructure of GaAs/Co_2_MnSi/[MnGa/Co_2_MnSi]_n_/MgO/CoFe/Pd and evaluated its TMR. The microstructure of this heterostructure was examined by cross-section high-resolution transmission electron microscopy (HRTEM). As shown in Fig. 5a, a periodical structure of multilayers was seen clearly. The thicknesses of the Co_2_MnSi and MnGa layers are about 0.9 nm and 4.5 nm respectively. The entire MTJ stack is single crystalline with an epitaxial relationship Co_2_MnSi (001) (110)//MnGa (001) (100), which was confirmed by *in-situ* reflection high-energy electron diffraction (RHEED) patterns. None of the layers is much below 1 nm thick, in contrast to other metal multilayers such as Co/Pt where the layer thickness are typically a few angstroms; as a result, the structure should be amenable to wafer-scale fabrication of spintronic devices.

**Figure 6:**
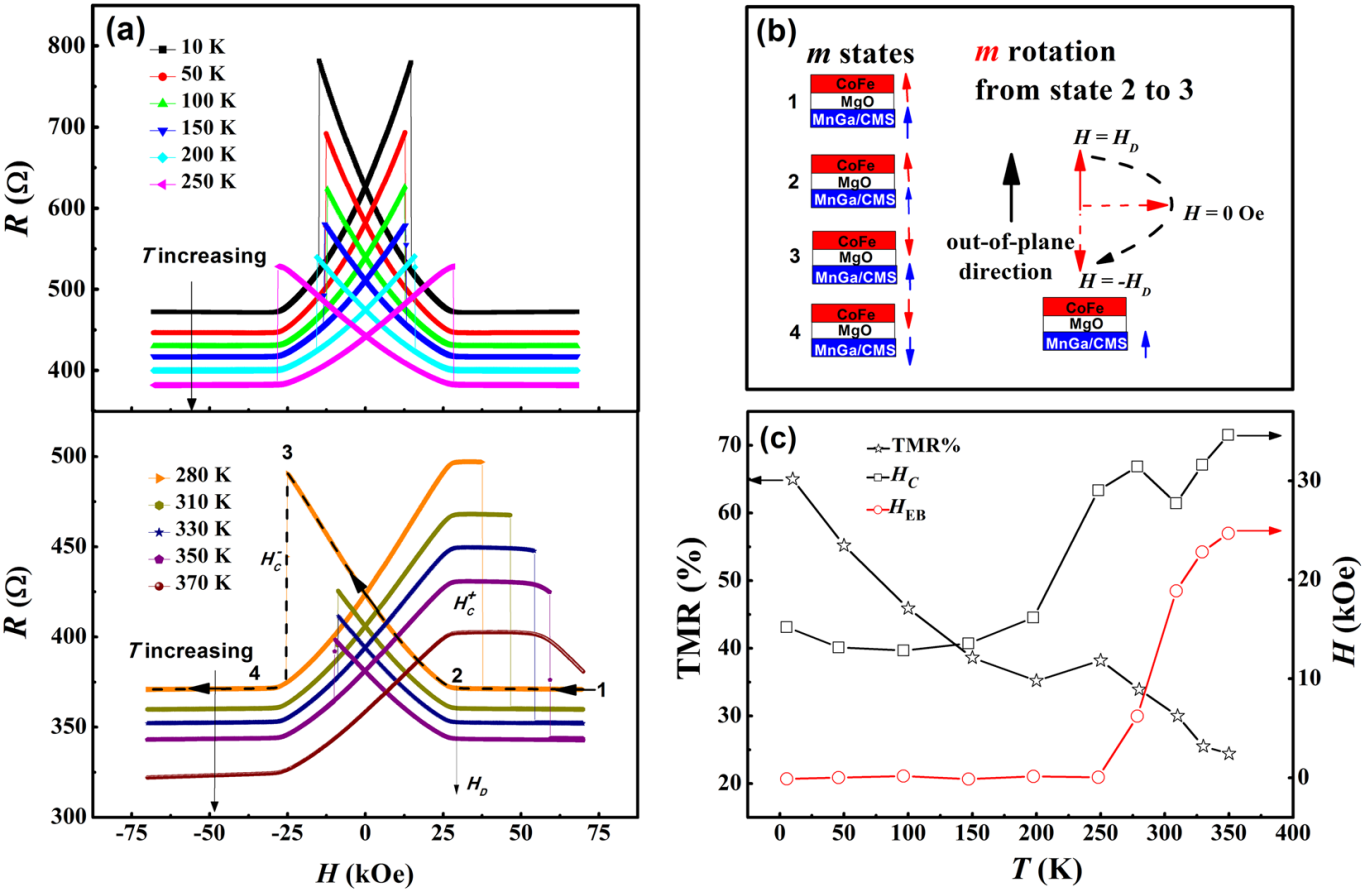
TMR in an MTJ with a [MnGa(4.5 nm)/Co_2_MnSi(0.9 nm)]_4_ reference electrode. (**a**) Resistance of the MTJ versus applied perpendicular magnetic field (*R*-*H*) measured at temperatures from 10 to 370 K. (**b**) Schematic depiction of the four different magnetic states for the MTJ in a down-sweep (positive to negative magnetic field: dash line and black arrows). The switchings between the magnetic states are marked by numbers 1 to 4. The magnetic moment rotation of the CoFe from state 2 to 3 is depicted separately. (**c**) TMR, coercivity *H*_c_, and the exchange bias field *H*_EB_ at different temperatures. The TMR is defined as $({R}_{{\rm{state}}3}-{R}_{{\rm{state}}1})/{R}_{{\rm{state}}1}$, *H*_c_ and *H*_EB_ are determined by $({H}_{{\rm{C}}}^{+}-{H}_{{\rm{C}}}^{-})/2$ and $({H}_{{\rm{C}}}^{+}+{H}_{{\rm{C}}}^{-})/2$, respectively.

The heterostructure is patterned into an MTJ device, as schematically shown in Fig. 5b, via photolithography and ion milling. The TMR of this superlattice-based MTJ was measured at different temperatures from 10 to 370 K, and the results are shown in Fig. 6a. The junction resistance versus perpendicular magnetic field (*R*-*H*) has a basic symmetrical butterfly shape at temperatures below 250 K, beyond which an asymmetry gradually develops. Figure 6b shows schematically the four different magnetic states corresponding to the TMR, with the magnetic switchings marked by numbers 1 to 4 for a down-sweep MR curve (positive to negative magnetic field); the magnetic moment rotation of the CoFe from state 2 to 3 is depicted, which results in a linear TMR response within its demagnetizing field (*H*_D_ to - *H*_D_). At 280 K and above, an asymmetry in the TMR becomes evident, clearly indicated by a decrease of ${H}_{{\rm{C}}}^{-}$ and increase of ${H}_{{\rm{C}}}^{+}$. The increase of ${H}_{{\rm{C}}}^{+}$ beyond + *H*_*D*_ leads to a null response of the junction resistance to increasing applied field (a plateau). At the highest measurement temperatures (350 K and 370 K), ${H}_{{\rm{C}}}^{+}$ is so high that in applied field beyond 59.3 kOe, the junction resistance *decreases* with increasing field. The prominent exchange bias evident in the TMR traces at high temperatures is unexpected since it is not observed in the AHE measurements of the unpatterned [MnGa(*t* nm)/Co_2_MnSi(1 nm)]_5_ superlattices (Fig. 4a). We surmise that the surprising effect in the MTJ originates from an *in-plane* EB coupling between the MnGa/Co_2_MnSi directly under the MTJ and that in the surrounding area defined by the ion milling. The initialization and evolution of such an exchange-biased state for a similar MTJ are presented in Section II of the Supplementary Information. The corresponding *R*-*H* curves at high temperatures (350 K and 300 K) following an unusual field *warming* process show pronounced asymmetries indicative of the in-plane exchange bias effect, with EB fields of 25 kOe and 17.5 kOe respectively.

Outside the junction area, the ion milling etches into the [MnGa/Co_2_MnSi]_4_ superlattice (Fig. 5b). The different thicknesses of the MnGa/Co_2_MnSi multilayer under the MTJ and outside of it result in different degrees of magnetic moment compensation. However, in the etched area, the magnetic compensation mechanism remains effective, which we verified by SQUID magnetometry measurements on a reference sample obtained by dry-etching a piece of the heterostructure for an MTJ without any resist mask during the ion milling step of the MTJ mesa fabrication. As presented in Section III of the Supplementary Information, the coercivity of the uniformly etched reference sample *increases* as temperature increases, and the *H*_*C*_ shows a negative correlation with the net areal magnetization of the sample; both are indicators of the magnetic compensation. Furthermore, the ion bombardment induced damages to the etched area may also alter its magnetization dynamics. Hence, the MnGa/Co_2_MnSi multilayers in the two different areas may well have different coercive fields (*H*_c_) with different temperature dependencies, which could lead to the unusual in-plane EB effect. As evident in the TMR curve, at 370 K neither *H*_c_ nor the EB field (*H*_EB_) is reached at an external magnetic field of 70 kOe. Microscopically, we speculate that EB stems from the exchange coupling at the boundary between the MnGa/Co_2_MnSi electrode directly under the MTJ and that in the surrounding area.

As shown in Fig. 6c, the TMR decreases from 65.0% to 24.4% with increasing temperature. The values are much lower than the TMR of Co_2_MnSi based MTJs, probably due to poor interfacial quality caused by large lattice mismatch between the superlattice electrodes and MgO barrier and/or diffusion of Mn atoms into the MgO barrier. Nevertheless, both *H*_c_ and *H*_EB_ *increase* with increasing temperature above 200 K. This feature is in good agreement with the trend revealed in the AHE measurements in a similar temperature range on a single [MnGa (4.5 nm)/Co_2_MnSi(1 nm)]_4_ superlattice. For the *H*_c_ versus temperature curve of the MTJ, there is a peak at 280 K, and *H*_c_ continue to rise after 310 K due to the dramatically increased EB field. It is worth noting that the unusual temperature dependence of the ${H}_{{\rm{EB}}}$ implies that the orientation of *H*_EB_ can be changed by a field warming process, which is distinct from the traditional procedure for building an exchange bias field in FM/AFM systems. These results indicate that this [MnGa(4.5 nm)/Co_2_MnSi(0.9 nm)]_4_ superlattice alone serves as a reference electrode in the perpendicular MTJ device with exceptional thermal and magnetic stability.

## Discussion

As is evidenced in intrinsic FIMs such as Tb_x_Co_1−x_, DyCo_5_, and Sm_0.972_Gd_0.028_Al_2_ *etc*., magnetic compensation is a most important condition for large exchange bias effect in FM/FIM multilayers; we have adopted this concept in the design and synthesis of an artificial perpendicularly-magnetized FIM. By using an ultrathin Co_2_MnSi layer and choosing the appropriate MnGa/Co_2_MnSi thickness ratio, we have engineered the compensation state of an AFM-coupled MnGa/Co_2_MnSi superlattice and realized a high compensation temperature well beyond 400 K. Hence a thermally robust, perpendicularly magnetized synthetic FIM was successfully demonstrated. Both the as-grown superlattice and the subsequently fabricated MTJ exhibit *H*_C_ which *increases* with increasing temperature, a strong indicator of compensation-induced exchange coupling. Meanwhile, the high spin polarization in Co_2_MnSi is also preserved as evidenced by the large TMR. In the lithographically patterned MTJ device, an unexpected exchange bias effect emerges, which is absent in a similar unpatterned superlattice. The effect is attributed to an in-plane EB effect between the MnGa/Co_2_MnSi multilayers under the tunnel junction and in its surrounding areas. This in-plane EB effect can reach tens of kOe, which is large enough for a variety of applications. It should be noted that the *H*_c_ and *H*_EB_ are impacted by the device fabrication process, to which much attention should be paid for optimal and consistent device performance. A similar strategy of compensation point engineering may be applicable to a broad set of AFM-coupled bilayers of a PMA hard ferromagnet and a thin soft FM layer, such as MnAl/Co^37^, MnGa/Co^21,22^, MnGa/Co_2_FeSi^38^.

In conclusion, a thermally robust PMA synthetic FIM with a high compensation temperature beyond 400 K was successfully realized in the AFM-coupled [MnGa/Co_2_MnSi]_n_ superlattices. A prototype device for high field sensing with a structure of GaAs/Co_2_MnSi/[MnGa/Co_2_MnSi]_n_/MgO/CoFe/Pd was fabricated which demonstrated high thermal and magnetic stability beyond 370 K and 70 kOe simultaneously. The design method of this artificial hard perpendicular FIM can be adapted to a variety of AFM-coupled multilayer systems as well as functional spintronic devices.

## Methods

### Growth and characterization

[Mn_2.9_Ga (*t*)/Co_2_MnSi(1 nm)]_5_ was epitaxially grown on GaAs (001) substrates at 250 °C with different Mn_2.9_Ga thicknesses (*t*_A_ = 3.75, 4.5, 5.6 nm) in a VG80 molecular-beam epitaxy system, and annealed *in-situ* at 250 °C for 10 minutes. The atomic ratio of Mn/Ga in the MnGa was calibrated to be about 2.9/1.0 by energy-dispersive spectrometry. For the MTJ structure, besides the periodical Mn_2.9_Ga/Co_2_MnSi bilayers, near the MgO barrier one more Co_2_MnSi layer was deposited for better TMR ratio. MgO was deposited by an electron-beam evaporator at room temperature, and the CoFe layer was deposited at 200 °C. The entire growth process was monitored *in-situ* by reflection high-energy electron diffraction (RHEED). The RHEED patterns showed 1/2-order superlattice reflections along the [110]_Co2MnSi_ direction, indicating a *L*2_1_ phase^39^. The MTJ device structures were characterized by cross-sectional high-resolution transmission electron microscopy (HRTEM, Tecnai G2 F30). The magnetic properties of the superlattices were characterized by SQUID magnetometry with a maximum applied field of ±5 T. The MTJ with a junction size of 50 × 50 μm^2^ and Hall devices with an active area of 100 × 300 μm^2^ were fabricated by UV lithography and ion milling processes. The transport properties were measured by a Quantum Design PPMS with an applied magnetic field up to 9 T using the four-terminal DC method with a current of 10 μA. More measurement and device information could be found in Supporting Information.

### Data availability

The data sets generated during measurements and/or analysed during the current study are available from the corresponding author upon request.

## Electronic supplementary material

Supplementary Information
