# Supplementary material for: Design and Synthesis of an Artificial Perpendicular Hard Ferrimagnet with High Thermal and Magnetic Field Stabilities
Source: Sci Rep. 2017 Dec 5;7:16990. doi: 10.1038/s41598-017-16761-z (PMC5717302; doi:10.1038/s41598-017-16761-z)
Supplement: Supplementary file 1 — Supplementary Information [file 41598_2017_16761_MOESM1_ESM.pdf]

Supplementary Information

Design and Synthesis of an Artificial Perpendicular Hard Ferrimagnet with High Thermal and Magnetic Field Stabilities

Jun Lu[1,2#], Siwei Mao[1,2#], Xupeng Zhao[1,2], Xiaolei Wang[1,2], Jian Liu[1,2], Jianbai Xia[1,2], Peng Xiong [3*] & Jianhua Zhao[1,2*]

1. *State Key Laboratory of Superlattices and Microstructures, Institute of Semiconductors, Chinese Academy of Sciences, P.O. Box 912, Beijing 100083, China*

2. *College of Materials Science and Opto-Electronic Technology, University of Chinese Academy of Sciences, Beijing 100049, China*

3. *Department of Physics, Florida State University, Tallahassee, FL 32306, USA*

*Correspondence and requests for materials should be addressed to J. H. Z. (email: jhzhao@red.semi.ac.cn) or P. X. (email: xiong@physics.fsu.edu)

[#]These authors contributed equally to the work.

Section I: The hysteresis loop of a single MnGa layer.

MnGa is a hard magnetic material with intrinsic perpendicular anisotropy. The hysteresis loop of a single MnGa (10 nm) layer is shown in **Figure S1**.

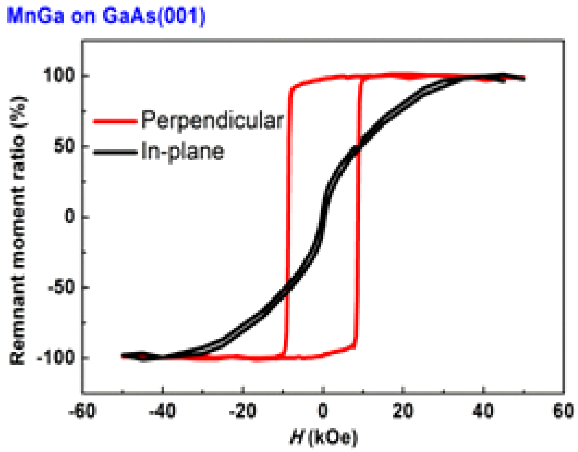

Figure S1. The hysteresis loop of a single MnGa layer.

Section II: Possible explanation of the asymmetric characteristic of the MTJ TMR-H curves.

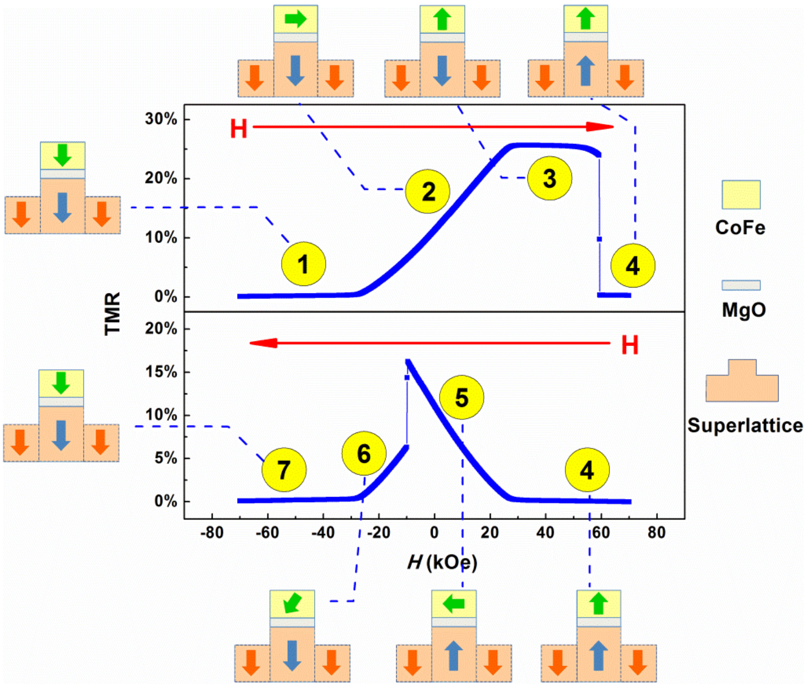

Figure S2. TMR-*H* curve (at 350 K) after the initialization of the starting state by a negative perpendicular field of -70 kOe at 10 K and then warming up to 350 K. The related magnetization state schematics of the MTJ are shown for different states in the subsequent field sweep.

The TMR-*H* curve for the MTJ develops a strong asymmetry as the temperature

increases (Fig. 6, main text), which suggests the emergence of an exchange-bias effect in the sample at high temperatures. Since no asymmetry is observed in the unpatterned film of the same structure as the MTJ, we attribute this phenomenon to an *in-plane* exchange coupling within the bottom [MnGa/Co_2MnSi]$_n$ electrode, between the portion directly under the MTJ (unprocessed) and that outside the MTJ (subjected to ion milling).

Figure S2 shows the TMR-*H* curve of an MTJ with apparent bias effect. The junction was initially magnetized in a negative perpendicular magnetic field of -70 kOe at 10 K, it was then warmed up to 350 K in this field. In taking the *R*-*H* curve, the field was swept from -70 kOe to +70 kOe (from state 1 to 4) and from +70 kOe to -70 kOe (state 4 to 7). After the initial magnetization process at low temperature, in state 1 the bottom electrode is uniformly magnetized in the negative direction, both directly under the MTJ and in the surrounding area. In state 2, the magnetic moment of the CoFe is gradually rotated from negative to positive direction, resulting in a linear MR, which plateaus in state 3. At about +60 kOe, the junction resistance exhibits a sharp drop to the low-resistance state, corresponding to the magnetization flip of the bottom electrode directly under the MTJ from negative to positive direction (state 4). The corresponding switching in reverse in the down-sweep occurs at a much lower field of -10 kOe. This asymmetry corresponds to an exchange bias field of 25 kOe. As illustrated in Fig. S1, we surmise that the exchange bias results from the pinning of the magnetic moment of the portion of the bottom electrode directly under the MTJ by that surrounding the junction (etched by ion milling).

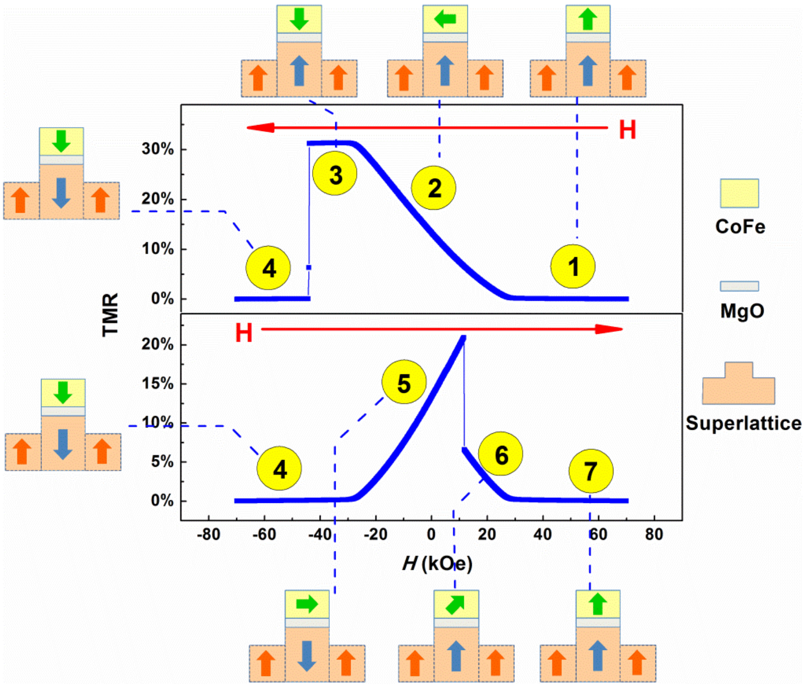

Figure S3. *TMR-H* curve (at 300 K) and the related magnetization state schematics of the same junction as in Figure 1. Here, the junction is initially magnetized in a positive perpendicular field of +70 kOe at 100 K, and then warmed up to 300 K.

In order to find support for our conclusion, we performed a comparative TMR-*H* measurement on the same junction. This time, the junction is initially magnetized in a positive perpendicular field of +70 kOe at 100 K, under which the magnetization of the bottom electrode is uniformly oriented in the positive direction. The junction is warmed up to 300 K in this field, followed by R-H measurement in field sweep from +70 kOe to -70 kOe (from state 1 to 4) and then from -70 kOe to +70 kOe (state 4 to 7), as shown in **Figure S3**. As expected, the switching field for the bottom electrode directly under the MTJ is now -45 kOe in the down-sweep and about +10 kOe in the up-sweep, indicating an exchange bias field of 17.5 kOe between the portion of the bottom electrode directly under the MTJ and that surrounding the junction.

Section III: Evolution of the H_C of the [MnGa/Co_2MnSi]$_4$ superlattice in the MTJ with temperature and net areal magnetization.

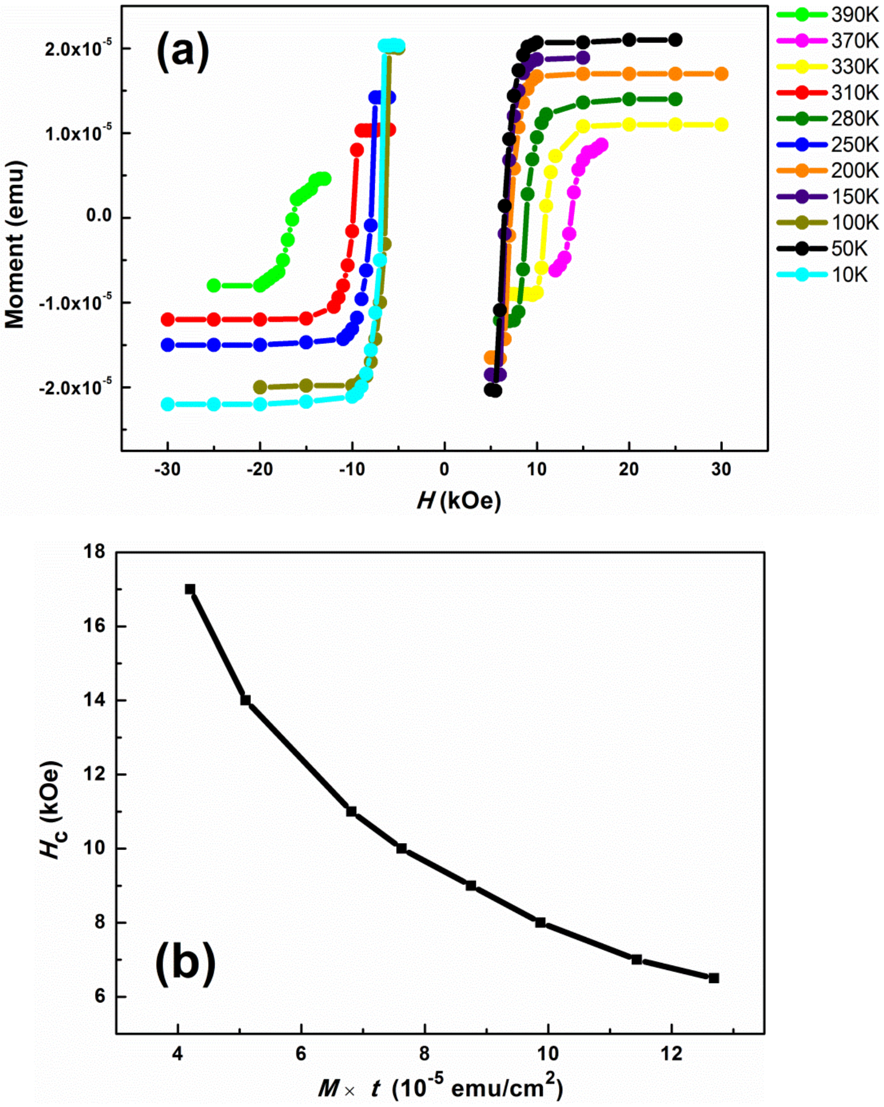

Figure S4. Magnetic properties of a control sample resembling the etched portion of the bottom MnGa/Co_2MnSi electrode outside the MTJ. **(a)** Superlattice *M-H* curves at various temperatures. **(b)** H_C dependence on the net areal magnetization.

Figure S4 (a) shows the relationship between H_C and the temperature of a $[MnGa/Co_2MnSi]_4$ sample which essentially represents the part of the bottom electrode surrounding the junction (subjected to ion milling) in the MTJ device discussed in Section I. The sample was obtained by dry-etching a piece of the heterostructure for the MTJ without any resist mask during the ion milling step of the MTJ mesa fabrication. As is evident in the data, the coercivity of the uniformly etched sample *increases* as temperature increases. Meanwhile, H_C shows a negative correlation with the net areal magnetization of the sample, which is clearly seen in **Figure S4 (b)**. Both of these phenomena are indicators that the magnetic moment compensation mechanism is still effective in this etched sample, and the compensation temperature of the sample exceeds 390 K.
